# Correction to “Sonochemical Formation of Fluorouracil Nanoparticles: Toward Controlled Drug Delivery from Polymeric Surfaces”

**DOI:** 10.1021/acsanm.4c06658

**Published:** 2024-12-07

**Authors:** Paulina Chytrosz-Wrobel, Monika Golda-Cepa, Piotr Kubisiak, Waldemar Kulig, Lukasz Cwiklik, Andrzej Kotarba

In the original version of this article on p. 4277 in Acknowledgments, there is an incomplete name of the source of financing. The correct Acknowledgments should be the following:

